# Analytic Expressions for Radar Sea Clutter WSSUS Scattering Functions †

**DOI:** 10.3390/e21090915

**Published:** 2019-09-19

**Authors:** Corey Cooke

**Affiliations:** Applied Technology, Inc., King George, VA 22485, USA; cdcooke21@students.tntech.edu

**Keywords:** airborne radar, radar clutter, radar signal processing, stochastic systems, time-varying systems, maximum entropy

## Abstract

Bello’s stochastic linear time-varying system theory has been widely used in the wireless communications literature to characterize multipath fading channel statistics. In the context of radar backscatter, this formulation allows for statistical characterization of distributed radar targets in range and Doppler using wide-sense stationary uncorrelated scattering (WSSUS) models. WSSUS models separate the channel from the effect of the waveform and receive filter, making it an ideal formulation for waveform design problems. Of particular interest in the radar waveform design community is the ability to suppress unwanted backscatter from the earth’s surface, known as clutter. Various methods for estimating WSSUS system functions have been studied in the literature, but to date no analytic expressions for radar surface clutter range-Doppler scattering functions exist. In this work we derive a frequency-selective generalization of the Jakes Doppler spectrum model, which is widely used in the wireless communications literature, adapt it for use in radar problems, and show how the maximum entropy method can be used to extend this model to account for internal clutter motion. Validation of the spectral and stationarity properties of the proposed model against a subset of the Australian Ingara sea clutter database is performed, and good agreement is shown.

## 1. Introduction

Random linear time-varying (LTV) system theory was first comprehensively described by Bello [2] and has widely been used in the wireless communications field ever since, particularly to model the multipath fading of mobile radio channels [3,4]. In particular, wide-sense stationary uncorrelated scattering (WSSUS) models, which are a subset of the category of random LTV systems, are the most common in the literature. As well, more recent work in this field has focused on nonstationary LTV communications channels [5,6,7], of which vehicle-to-vehicle (V2V) communication is a prime example [8]. A separate set of models that are distinct, but can be related to Bello’s LTV theory are the Clarke/Jakes Doppler spectrum class of models [9,10], originally only applicable for flat fading (i.e., where the symbol time is much larger than the multipath delay spread). This model is also ubiquitous in the wireless communications literature, and many extensions have been proposed, such as for varying geometries [11] and more accurate fading statistics [12]. It is also commonly coupled with Bello’s LTV theory (which is general enough to model frequency-selective fading where the multipath delay spread is much larger than the symbol duration) to model the Doppler component of multipath scattering [13]. Empirical [14] and analytic [15] expressions characterizing range-Doppler spreading of communication channels using LTV theory are commonplace.

Outside of communications applications, random LTV system models have been primarily used for sonar target detection and waveform design problems [16,17,18], although a few recent papers have applied this method to radar target detection problems as well [19,20]. Much of the literature on the subject treats the problem agnostically, treating radar and sonar as the same problem [21,22,23,24,25,26]. In both radar and sonar, the goal is to detect (usually) man-made targets of interest while suppressing thermal noise, which is internal to the receiver, and clutter, which are unwanted signal-dependent returns from the natural environment. Waveform design problems expressed in this form require characterization of the delay-Doppler “scattering functions” of the target and clutter, and the result is an optimized transmit waveform/receive filter pair.

In addition to waveform design problems, clutter Doppler spectrum characterization is useful for optimized moving target indication (MTI) and space-time adaptive processing (STAP) filtering, which optimize the receiver only. Specifically, the range-Doppler scattering function can be used to predict pulse-to-pulse correlations in the clutter return to suppress it using eigenfilter techniques [27,28]. An up-to-date summary of recent work on Doppler spectrum modeling can be found in [29]. Most papers on Doppler spectrum modeling do not use Bello’s LTV formulation; the effect of the transmit waveform, measurement system, and clutter are usually combined. This is appropriate if the goal is to generate realistic clutter samples for simulation purposes or if the goal is to design an MTI/STAP filter because the processing is done on receive. However, waveform design requires the clutter to be partitioned separately from the transmit signal, which creates a motivation to “translate” these Doppler spectrum results to the “language” of random LTV system theory so that these Doppler spectrum models can be utilized in other problem domains.

Because scattering function estimation is a fundamental component of waveform design problems, it is a topic that has been studied for decades [30,31,32,33,34,35,36,37,38,39,40,41]. However, analytic expressions for scattering functions of sea clutter for airborne radars have not been reported in the literature except for some initial work by the author [1,42,43]. In this work we derive such analytic expressions for the case when localized internal clutter motion (ICM) is small relative to radar platform motion using a frequency-selective version of the Jakes model. Extensions to this model are then proposed to connect previous work on Doppler spectrum modeling to random LTV theory. As well, we demonstrate how an estimation of spatially-local ICM spectra can be cast as a probability density estimation problem, for which solutions can be found using the Jaynes maximum entropy (MaxEnt) method [44] and directional statistics [45]. The spectrum prediction method is then validated against a subset of the Australian Ingara medium grazing angle clutter dataset [46], and good agreement is shown.

## 2. Results

### 2.1. Mathematical Background

For a monostatic radar of arbitrary polarization, the baseband backscattered signal y(t) will be modeled as the response of an LTV system h(τ,t) to a transmitted signal x(t):

(1) y(t)=∫h(τ,t)x(t−τ)dτ+w(t),

where τ represents downrange delay (i.e., “fast time”), *t* is absolute time (“slow time”), and w(t) is thermal noise. In the literature, h(τ,t) is commonly referred to as the time-varying impulse response. In this work, *h* will include the transceiver, antenna, atmospheric propagation, and backscattering environment, so x(t) is essentially the output of the transmit D/A, and y(t) is the input to the receive A/D, prior to matched filtering. Polarization considerations are contained in the antenna pattern and surface scattering radar cross-section (RCS). Variations in h(τ,t) with respect to *t* represent channel-induced Doppler shifts. In the case where the entire scenario is static, h(τ,t)=h(τ) (i.e., *h* is LTI), and the integral in Equation (1) reduces to a convolution. In applying the LTV model to a radar system, we use the following assumptions:

- The LTV impulse response h(τ,t) is only valid over a single coherent processing interval (CPI) of *N* transmitted pulses
- Over a single CPI, the range to target(s) is roughly constant
- Relative motion produces a Doppler shift on the carrier only and does not introduce time dilation of the pulse. The conditions for this assumption to be valid are that $BT<0.1c/\dot{r}$, where BT is the time-bandwidth product of the waveform and $\dot{r}$ is the maximum range rate [47] (p. 382).

We will model h(τ,t) as a wide-sense stationary uncorrelated scattering (WSSUS) process characterized by a set of related correlation functions and show how these are a function of sensor geometry, system parameters, and environmental conditions. WSSUS processes, which are a subset of all random LTV systems, are the most widely used model in channel characterization problems [3,13]. In the following sections, we will illustrate the properties of WSSUS processes and validate the core assumptions for the radar sea clutter problem.

### 2.2. WSSUS Processes

To apply the WSSUS assumption to a radar scattering environment, we will express the impulse response in the following form:

(2) $$h\left(\tau ,t\right)=F_{\rho }^{-1}\left[\eta \left(\tau ,\rho \right)\right]=\int_{-\infty }^{\infty }\eta \left(\tau ,\rho \right)e^{j2\pi \rho t}d\rho$$

where $F^{-1}$ is the inverse Fourier operator, ρ is Doppler shift, and η(τ,ρ) is known as the “delay-Doppler spread function” [13,31]. This function is a stochastic description of the range-Doppler map of the targets. Substituting Equation (2) into Equation (1) yields:

(3) $$y\left(t\right)=\int_{-\infty }^{\infty }\int_{-\infty }^{\infty }\eta \left(\tau ,\rho \right)x\left(t-\tau \right)e^{j2\pi \rho t}d\rho d\tau +w\left(t\right).$$

In Equation (3) it is clear to see that y(t) can be expressed as the superposition of delayed (by τ), frequency-shifted (by ρ), and scaled (by the complex gain η(τ,ρ)dτdρ) copies of x(t). It should be noted that this definition is general enough to include all scatterers in the radar’s field of view—clutter as well as useful targets. In this case the overall delay-Doppler spreading function is:

(4) $$\eta_{\mathrm{total}}=\eta_{\mathrm{targets}}+\eta_{\mathrm{clutter}},$$

due to the linearity of the system.

To characterize the behavior of *h* as a stochastic process, we will assume it is zero mean and compute its second-order statistics:

(5) $$R_{h}\left(\tau ,\tau^{\prime },t,t+\Delta t\right)=E\left[h^{*}\left(\tau ,t\right)h\left(\tau^{\prime },t+\Delta t\right)\right].$$

Here will we apply the two fundamental assumptions of a WSSUS system: (1) The system is wide-sense stationary (WSS), implying that the autocorrelation depends only on Δt along the *t* axis, and (2) the scattering at different lags τ are uncorrelated (US):

(6) $$\begin{array}{cc} E\left[h^{*}\left(\tau ,t\right)h\left(\tau^{\prime },t+\Delta t\right)\right] & =R_{h}\left(\tau ,\tau^{\prime },\Delta t\right)\\ & =A_{h}\left(\tau ,\Delta t\right)\delta \left(\tau -\tau^{\prime }\right). \end{array}$$

The function $A_{h}\left(\tau ,\Delta t\right)$ is known as the system correlation function [3,48]. By taking the Fourier transform of $A_{h}\left(\tau ,\Delta t\right)$ with respect to Δt, we can view the Doppler spectrum of the return as a function of delay, referred to in the literature as the “scattering function” $S_{h}\left(\tau ,\rho \right)$:

(7) $$S_{h}\left(\tau ,\rho \right)=F_{\Delta t}\left[A_{h}\left(\tau ,\Delta t\right)\right].$$

The scattering function can be thought of as the true range-Doppler map of the scattering environment independent of the waveform used to probe it. It can also be shown that the autocorrelation function of the delay-Doppler spread function η for a WSSUS system is:

(8) $$E\left[\eta^{*}\left(\tau ,\rho \right)\eta \left(\tau^{\prime },\rho^{\prime }\right)\right]=S_{h}\left(\tau ,\rho \right)\delta \left(\tau -\tau^{\prime }\right)\delta \left(\rho -\rho^{\prime }\right).$$

It is sometimes easier to work with this form when deriving new expressions. This result shows that under a WSSUS model, the scattering not only at different ranges, but also different Doppler shifts are uncorrelated.

**Figure 1:**
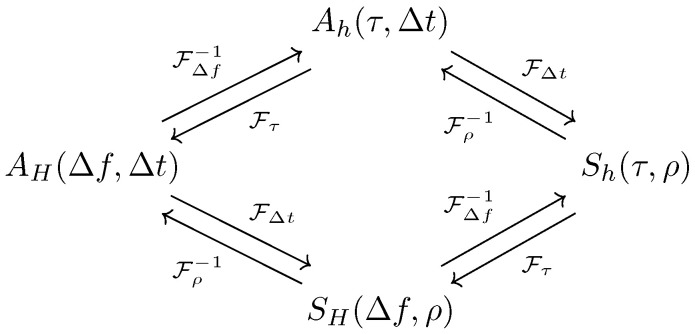
Wide-sense stationary uncorrelated scattering (WSSUS) system function relationships. Knowledge of any one of these functions grants complete knowledge of the second-order statistics of the system.

The full set of WSSUS system functions are shown in Figure 1, all of which are related by Fourier transforms of the different temporal variables. Knowledge of any one of these functions is a sufficient description of the second-order statistics of the system. These functions are standard tools in wireless communication channel modeling, and the diagram shown in Figure 1 can be found in virtually any textbook on the subject [3].

Note that nowhere in this discussion have we specified a distribution for the samples of h(τ,t). If we assume that *h* is a circularly-symmetric complex Gaussian random process, then its distribution is completely specified by the WSSUS system functions described previously, and the magnitude envelope |y| of the return will be Rayleigh distributed [3]. However, in many cases a distribution with heavier tails, such as the Weibull or K distribution, is more appropriate, particularly for low grazing angles or when the radar can resolve individual sea spikes [49]. The results derived in the remainder of this paper, however, are valid regardless of the distribution of *h*. Note, however, that if *h* is non-Gaussian, higher-order statistics (third-order and above) are required to uniquely specify all of its properties.

### 2.3. Simulation Geometry

**Figure 2:**
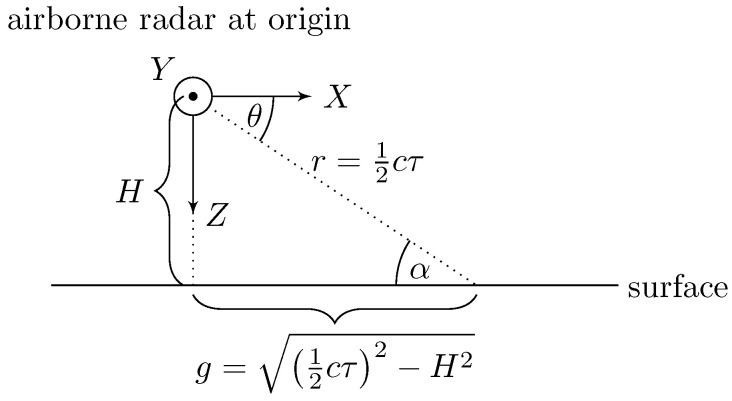
Flat earth geometry in the *X*-*Z* plane. Because the goal is to ultimately produce a range-Doppler map, we need to express all quantities in terms of downrange delay τ and absolute time *t*.

The simulation geometry is shown in Figure 2, where the airborne radar is at an altitude *H* above the surface traveling with velocity $v={\left[v_{X},v_{Y},v_{Z}\right]}^{T}$. We will assume a coordinate system fixed to the phase center of the radar antenna, such that the antenna is at the origin, the *X*-axis is pointed parallel to the surface and in the plane of symmetry of the aircraft, the *Y*-axis is pointed out the right side of the aircraft and parallel to the surface, and the *Z*-axis is pointing down. In this work, we will assume that v always lies in the *X*-*Z* plane (i.e., no crabbing). Because we are seeking to model the scattering characteristics as a function of *t* and τ, we will need to express all spatial quantities in terms of these temporal variables.

**Figure 3:**
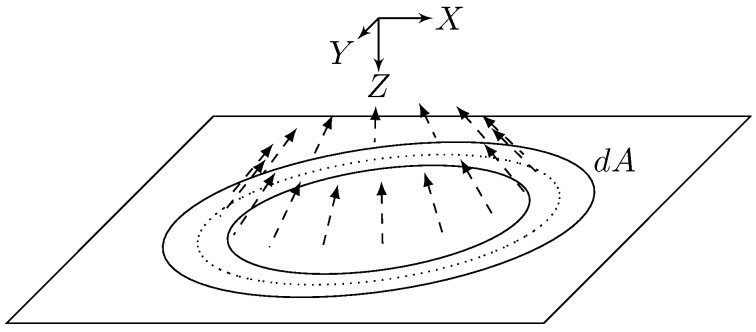
Return from a single infinitesimal isorange ring on the sea surface. The autocorrelation function of the impulse response of this ring is computed at delay $\tilde{\tau }$, then the result is integrated over all $\tilde{\tau }$ to get the total autocorrelation as a function of delay τ and time offset Δt.

We will model the radar signal using an approach similar to Barrick [50,51], i.e., as a spherical wave emanating from the source located at the origin as is shown in Figure 3. Each small segment of the wave reflects off of successive isorange rings on the sea surface; the total return is therefore the superposition of the returns from each isorange ring. The incremental power gain as a function of delay can be obtained using the radar range equation:

(9) $$\frac{dP_{R}}{P_{T}}=\frac{G^{2}\left(\phi \left(\tau ,\rho \right),\theta \left(\tau \right)\right)\lambda^{2}\sigma^{0}\left(\alpha \left(\tau \right)\right)dA\left(\tau \right)}{{\left(4\pi \right)}^{3}{\left(\frac{1}{2}c\tau \right)}^{4}}$$

where:

- $P_{T}$ = transmit power
- $dP_{R}$ = incremental received power from isorange ring at delay τ
- ϕ,θ = azimuth and depression angles relative to platform
- α = grazing angle, which equals θ in a flat earth model
  $\Rightarrow \alpha =\theta =\sin^{-1}\left(H/\left(\frac{1}{2}c\tau \right)\right)$
- G(ϕ,θ) = one-way antenna power gain
- λ = carrier wavelength
- $\sigma^{0}=\sigma^{0}\left(\alpha ,\lambda ,\mathrm{sea}\;\mathrm{state},\ldots \right)=$ surface normalized radar cross section (NRCS)
- dA = $\frac{\pi }{2}c^{2}\tau d\tau$ = incremental area of isorange ring at delay τ.

### 2.4. WSSUS System Function Derivations

To create a model for the scattering function $S_{h}\left(\tau ,\rho \right)$, we need to characterize the signal return as a function of delay and Doppler. To do so, we will consider the Doppler spectrum generated from the backscatter from a single isorange ring and then apply the principle of superposition to obtain the total response from all ranges. The approach taken in this section can be considered a generalization of the Clarke model for flat fading [9,12], applied to modeling radar surface clutter.

We will assume that the impulse response from this ring is the superposition of returns from *N* equiangular patches in azimuth with random amplitudes and phases. We will also assume that the scatterers on this ring are located at delay $\tilde{\tau }$ and write the incremental impulse response of this thin ring as follows:

(10) $$dh\left(\tau ,t;\tilde{\tau }\right)=\delta \left(\tau -\tilde{\tau }\right)\sum_{n=0}^{N-1}da\left(\phi_{n}\right)e^{j(2\pi \rho \left(\phi_{n}\right)t+\gamma_{n})}$$

where $\phi_{n}=2\pi n/N$ is the azimuth angle to patch *n*, $da(\phi_{n})$ is the infinitesimal amplitude gain at angle $\phi_{n}$, $\rho (\phi_{n})$ is the Doppler shift at angle $\phi_{n}$, and $\gamma_{n}$ is a random phase shift. The incremental autocorrelation function of the impulse response at $\tilde{\tau }$ is then given by:

(11) $$\begin{array}{cc} dR_{h}\left(\tau ,\tau^{\prime },t,t+\Delta t;\tilde{\tau }\right) & =E\left[dh^{*}\left(\tau ,t;\tilde{\tau }\right)dh\left(\tau^{\prime },t+\Delta t;\tilde{\tau }\right)\right]\\ & =\delta \left(\tau -\tilde{\tau }\right)\delta \left(\tau^{\prime }-\tilde{\tau }\right)\sum_{m,n}E\left[da\left(\phi_{m}\right)da\left(\phi_{n}\right)e^{j[2\pi \left(\rho \left(\phi_{n}\right)\left(t+\Delta t\right)-\rho \left(\phi_{m}\right)t\right)+\gamma_{n}-\gamma_{m}]}\right]. \end{array}$$

We will also assume that the scatterer amplitudes $da(\phi_{n})$ are mutually independent, and we will assume that the amplitude $da(\phi_{n})$ is independent of the Doppler shift $\rho (\phi_{n})$, therefore:

(12) $$\begin{array}{cc} dR_{h}\left(\tau ,\tau^{\prime },t,t+\Delta t;\tilde{\tau }\right) & =\delta \left(\tau -\tilde{\tau }\right)\delta \left(\tau^{\prime }-\tilde{\tau }\right)\sum_{n=0}^{N-1}E\left[|da\left(\phi_{n}\right){|}^{2}\right]E\left[e^{j2\pi \rho (\phi_{n})\Delta t}\right]\\ & =dR_{h}\left(\tau ,\tau^{\prime },\Delta t;\tilde{\tau }\right) \end{array}$$

Note that $E\left[|da\left(\phi_{n}\right){|}^{2}\right]$ is simply the backscattered power gain from scatterer *n*, therefore by using Equation (9) and scaling it to account for the fact that the surface area of each patch is smaller by a factor of 1/N, we obtain:

(13) $$\begin{array}{cc} E\left[|da\left(\phi_{n}\right){|}^{2}\right] & =\frac{1}{N}\frac{dP_{R}}{P_{T}}\\ & =\frac{\lambda^{2}\sigma^{0}d\tilde{\tau }}{8\pi^{2}c^{2}{\tilde{\tau }}^{3}}\frac{1}{N}G^{2}\left(n\Delta \phi ,\theta \right). \end{array}$$

Note that N=2π/Δϕ, where Δϕ is the angular spacing between patches, which upon substituting in Equation (13) yields:

(14) $$E\left[|da\left(\phi_{n}\right){|}^{2}\right]=\frac{\lambda^{2}\sigma^{0}d\tilde{\tau }}{16\pi^{3}c^{2}{\tilde{\tau }}^{3}}G^{2}\left(n\Delta \phi ,\theta \right)\Delta \phi .$$

Combining this with Equation (12) and taking the limit as N→∞ yields

(15) $$\begin{array}{cc} dR_{h}\left(\tau ,\tau^{\prime },\Delta t;\tilde{\tau }\right) & =\delta \left(\tau -\tilde{\tau }\right)\delta \left(\tau^{\prime }-\tilde{\tau }\right)\frac{\lambda^{2}\sigma^{0}d\tilde{\tau }}{16\pi^{3}c^{2}{\tilde{\tau }}^{3}}\cdot \lim_{N\to \infty }\sum_{n=0}^{N-1}G^{2}\left(n\Delta \phi ,\theta \right)E\left[e^{j2\pi \rho (\phi_{n})\Delta t}\right]\Delta \phi \\ & =\delta \left(\tau -\tilde{\tau }\right)\delta \left(\tau^{\prime }-\tilde{\tau }\right)\frac{\lambda^{2}\sigma^{0}d\tilde{\tau }}{16\pi^{3}c^{2}{\tilde{\tau }}^{3}}\int_{\langle 2\pi \rangle }G^{2}\left(\phi ,\theta \right)E\left[e^{j2\pi \rho_{\phi ,\theta }\Delta t}\right]d\phi . \end{array}$$

Note that the expression $E\left[e^{j2\pi \rho_{\phi ,\theta }\Delta t}\right]$ is the characteristic function of the random angular frequency $2\pi \rho_{\phi ,\theta }$[52]. In this work, we will define the characteristic function k(Δt|ϕ,θ) as:

(16) $$\begin{array}{cc} k(\Delta t|\phi ,\theta ) & =\int p\left(\rho |\phi ,\theta \right)e^{j2\pi \rho \Delta t}d\rho \\ & =F_{\rho }^{-1}\left[p(\rho |\phi ,\theta )\right], \end{array}$$

where p(ρ|ϕ,θ) is the probability density function (pdf) of the random frequency $\rho_{\phi ,\theta }$. Substituting this expression in Equation (15) yields:

(17) $$\begin{array}{cc} dR_{h}\left(\tau ,\tau^{\prime },\Delta t;\tilde{\tau }\right) & =\delta \left(\tau -\tilde{\tau }\right)\delta \left(\tau^{\prime }-\tilde{\tau }\right)\frac{\lambda^{2}\sigma^{0}d\tilde{\tau }}{16\pi^{3}c^{2}{\tilde{\tau }}^{3}}\cdot \lim_{N\to \infty }\sum_{n=0}^{N-1}G^{2}\left(n\Delta \phi ,\theta \right)E\left[e^{j2\pi \rho_{n}\Delta t}\right]\Delta \phi \\ & =\delta \left(\tau -\tilde{\tau }\right)\delta \left(\tau^{\prime }-\tilde{\tau }\right)\frac{\lambda^{2}\sigma^{0}d\tilde{\tau }}{16\pi^{3}c^{2}{\tilde{\tau }}^{3}}\cdot \int_{\langle 2\pi \rangle }G^{2}\left(\phi ,\theta \right)k\left(\Delta t|\phi ,\theta \right)d\phi \end{array}$$

One interpretation of the function p(ρ|ϕ,θ) that is used in the wireless communications literature is that the pdf of the random Doppler shift can be thought of as a normalized power spectral density (PSD) [3,8]. Thus if one has some model of the local Doppler spectrum, this information is accounted for in Equation (16).

To obtain the autocorrelation function for all delays τ we will use the US property that the return from each isorange ring $\tilde{\tau }$ is uncorrelated and thus we can integrate over $\tilde{\tau }$ to obtain the total response:

(18) $$\begin{array}{c} R_{h}\left(\tau ,\tau^{\prime },\Delta t\right)=\int dR_{h}\left(\tau ,\tau^{\prime },\Delta t;\tilde{\tau }\right)\\ =\delta \left(\tau -\tau^{\prime }\right)\frac{\lambda^{2}\sigma^{0}}{16\pi^{3}c^{2}\tau^{3}}\int_{\langle 2\pi \rangle }G^{2}\left(\phi ,\theta \right)k\left(\Delta t|\phi ,\theta \right)d\phi . \end{array}$$

It is clear from Equation (18) that $A_{h}\left(\tau ,\Delta t\right)$ is therefore:

(19) $$A_{h}\left(\tau ,\Delta t\right)=\frac{\lambda^{2}\sigma^{0}}{16\pi^{3}c^{2}\tau^{3}}\int_{\langle 2\pi \rangle }G^{2}\left(\phi ,\theta \right)k\left(\Delta t|\phi ,\theta \right)d\phi .$$

To find the clutter scattering function $S_{h}\left(\tau ,\rho \right)$, we take the Fourier transform of $A_{h}\left(\tau ,\Delta t\right)$ with respect to Δt:

(20) $$\begin{array}{cc} S_{h}\left(\tau ,\rho \right) & =F_{\Delta t}\left[A_{h}\left(\tau ,\Delta t\right)\right]\\ & =\frac{\lambda^{2}\sigma^{0}}{16\pi^{3}c^{2}\tau^{3}}\int_{\langle 2\pi \rangle }G^{2}\left(\phi ,\theta \right)\left(F_{\Delta t}F_{\rho }^{-1}p\left(\rho |\phi ,\theta \right)\right)d\phi \\ & =\frac{\lambda^{2}\sigma^{0}}{16\pi^{3}c^{2}\tau^{3}}\int_{\langle 2\pi \rangle }G^{2}\left(\phi ,\theta \right)p\left(\rho |\phi ,\theta \right)d\phi \end{array}$$

The expression in Equation (20) is significant because it gives an analytical expression for the full clutter spectrum, not just the mainlobe clutter, for any antenna pattern and provides a mechanism for supplying a priori information about the local Doppler spectra as a function of look angle. In this integral it can be seen that the antenna pattern has the effect of performing a weighted average of the local Doppler spectra over azimuth.

### 2.5. Important Special Cases

In this section, we will use Equation (20) to derive analytic expressions for the scattering function for several useful cases.

#### 2.5.1. No ICM

In the degenerate case where there is no ICM, i.e., the surface motion is small relative to the platform motion, then the localized Doppler shifts $\rho_{\phi ,\theta }$ are deterministic and only depend on the look angle relative to platform motion. This means that the local Doppler spectra are each just an impulse, and the scattering function Equation (20) reduces to [42]:

(21) $$\begin{array}{cc} S_{h}^{(\mathrm{no}}\;\mathrm{ICM})\left(\tau ,\rho \right) & =\frac{\lambda^{2}\sigma^{0}}{16\pi^{3}c^{2}\tau^{3}}\int_{\langle 2\pi \rangle }G^{2}\left(\phi ,\theta \right)\delta \left(\rho -\beta \left(\phi ,\theta \right)\right)d\phi \\ & =\left\{\begin{array}{cc} \frac{\lambda^{2}\sigma^{0}\left(\alpha \left(\tau \right)\right)}{16\pi^{3}c^{2}\tau^{3}}\frac{G^{2}\left(\phi^{\prime },\theta \right)+G^{2}\left(-\phi^{\prime },\theta \right)}{\sqrt{{\left(\rho_{X}^{\prime }\left(\tau \right)\right)}^{2}-{\left(\rho -\rho_{Z}^{\prime }\left(\tau \right)\right)}^{2}}}, & |\rho -\rho_{Z}^{\prime }\left(\tau \right)|<\rho_{X}^{\prime }\left(\tau \right)\\ 0, & \mathrm{else}, \end{array}\right. \end{array}$$

where the nominal Doppler shift β(ϕ,θ) due to platform motion is given as:

(22) $$\beta \left(\phi ,\theta \right)=\rho_{X}\cos\theta \cos\phi +\rho_{Z}\sin\theta ,$$

where $\rho_{X}=2v_{X}/\lambda$ and $\rho_{Z}=2v_{Z}/\lambda$ are the Doppler contributions due to motion in the X−Z plane, $\rho_{X}^{\prime }\left(\tau \right)=\rho_{X}\cos\left(\theta \left(\tau \right)\right)$ and $\rho_{Z}^{\prime }\left(\tau \right)=\rho_{Z}\sin\left(\theta \left(\tau \right)\right)$ are the effective Doppler contributions in the observation direction, and $\phi^{\prime }\left(\tau ,\rho \right)=\arccos\left(\left(\rho -\rho_{Z}^{\prime }\left(\tau \right)\right)/\rho_{X}^{\prime }\left(\tau \right)\right)$ is the nominal azimuth angle. The result in Equation (21) is a frequency-selective generalization of the Clarke/Jakes spectrum for airborne radar geometries [9,10].

**Figure 4:**
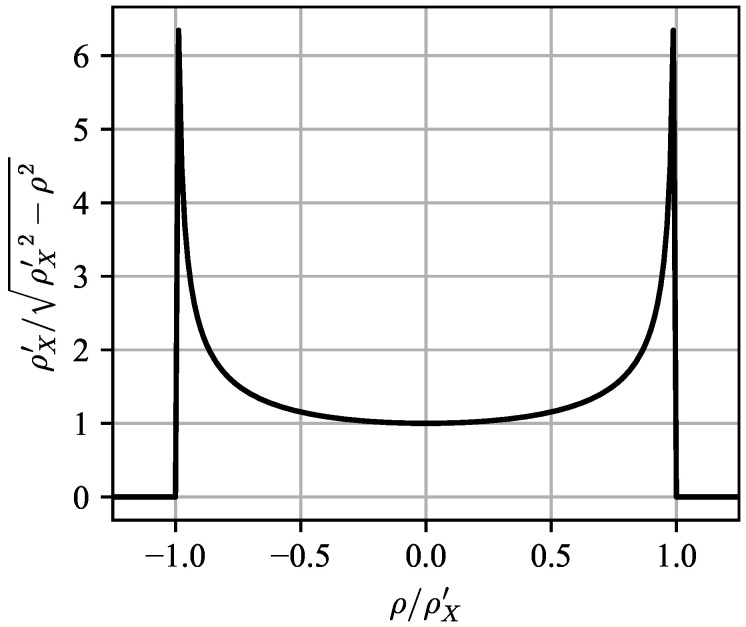
Constant-τ cut of the no-internal clutter motion (ICM) scattering function Equation (21) with the antenna pattern removed (i.e., G=1) and no vertical motion ($\rho_{Z}^{\prime }=0$). The true scattering function will be windowed by the antenna pattern to focus on a specific region of ρ—a side-looking antenna will be focused near ρ=0, whereas a nose-aspect antenna will be focused near $\rho =\rho_{X}^{\prime }$.

A constant-τ cut of Equation (21) without the antenna pattern is plotted in Figure 4, with G=1 and $\rho_{Z}^{\prime }=0$. The singularity of the scattering function at $\rho -\rho_{Z}^{\prime }\left(\tau \right)\approx \rho_{X}^{\prime }\left(\tau \right)$ is extremely critical for modeling nose-aspect clutter, as it serves to narrow the Doppler spread of the antenna pattern. For side-looking antennas, however, the singularities are irrelevant as the Jakes spectrum is flat near $|\rho -\rho_{Z}^{\prime }\left(\tau \right)|\approx 0$.

#### 2.5.2. Side-Looking Antenna, Level Flight Path

In many airborne radars, the antenna broadside is pointed at ϕ=−π/2. In this case, we will create a new angular variable ψ=ϕ+π/2 to represent angular deviation from broadside. The antenna pattern and local Doppler spectrum with respect to this coordinate will be denoted $\tilde{G}$ and $\tilde{p}$, respectively. If we assume a level flight path (i.e., $\rho_{Z}=0$), then:

(23) $$\begin{array}{cc} \beta (\phi ,\theta ) & =\rho_{X}\cos\theta \cos\phi \\ & =\rho_{X}\cos\theta \sin\psi \\ \approx & \left(\rho_{X}\cos\theta \right)\psi =\rho_{X}^{\prime }\psi , \end{array}$$

which, for an antenna with beamwidth of less than 20°, is a very good approximation. Substituting into Equation (20) to compute the scattering function yields:

(24) $$\begin{array}{cc} S_{h}^{\left(\mathrm{side}\right)}\left(\tau ,\rho \right) & =\frac{\lambda^{2}\sigma^{0}}{16\pi^{3}c^{2}\tau^{3}}\int_{-\pi }^{\pi }{\tilde{G}}^{2}\left(\psi ,\theta \right)\tilde{p}\left(\rho |\psi ,\theta \right)d\psi \\ \approx & \frac{\lambda^{2}\sigma^{0}}{16\pi^{3}c^{2}\tau^{3}}\int_{-\infty }^{\infty }{\tilde{G}}^{2}\left(\psi ,\theta \right)\tilde{p}\left(\rho |\psi ,\theta \right)d\psi \\ & =\frac{\lambda^{2}\sigma^{0}}{16\pi^{3}c^{2}\tau^{3}}\frac{1}{\rho_{X}^{\prime }}\int_{-\infty }^{\infty }{\tilde{G}}^{2}\left(\beta /\rho_{X}^{\prime },\theta \right)\tilde{p}\left(\rho |\beta /\rho_{X}^{\prime },\theta \right)d\beta , \end{array}$$

where the second equality is due to the fact that the antenna gain is nearly zero near the edges of the angular limit, so extending the limits of the integral to infinity does not change the result, and the third equality converts the integral from the angular domain to the Doppler domain. If we simplify the integrals by defining $\underline{G}\left(\beta |\tau \right)=\tilde{G}\left(\beta /\rho_{X}^{\prime }\left(\tau \right),\theta \left(\tau \right)\right)$ and $\underline{p}\left(\rho |\beta ,\tau \right)=\tilde{p}\left(\rho |\beta /\rho_{X}^{\prime }\left(\tau \right),\theta \left(\tau \right)\right)$, which are just the antenna pattern and local Doppler spectrum projected into delay-Doppler space, we can see that the scattering function is just a linear transformation of the antenna pattern with the local Doppler spectrum acting as the kernel function:

(25) $$S_{h}^{\left(\mathrm{side}\right)}\left(\tau ,\rho \right)=\frac{\lambda^{2}\sigma^{0}}{16\pi^{3}c^{2}\tau^{3}}\frac{1}{\rho_{X}^{\prime }}\int_{-\infty }^{\infty }{\underline{G}}^{2}\left(\beta |\tau \right)\underline{p}\left(\rho |\beta ,\tau \right)d\beta .$$

Note that $\underline{p}\left(\rho |\beta ,\tau \right)$ is the Doppler spectra observed by a moving platform, meaning that its center frequency is being modulated by the platform Doppler shift β(ϕ,θ). If we assume that $\underline{p}$ does not change much relative to β, which is a reasonable assumption due to the fact that radar antennas usually have a very small azimuthal beamwidth, we can express $\underline{p}$ in terms of a “baseband” Doppler spectrum, i.e., the Doppler spectrum caused only by ICM that would be seen by a stationary radar with an infinitesimal beamwidth. The ICM spectrum is usually what is discussed in papers on Doppler spectrum modeling (e.g., [28,29,53]). We will denote this quantity as $\underline{b}\left(\rho |\tau \right)$ and note that under the narrow beamwidth assumption, $\underline{p}\left(\rho |\beta ,\tau \right)\approx \underline{b}\left(\rho -\beta |\tau \right)$, therefore:

(26) $$\begin{array}{cc} S_{h}^{\left(\mathrm{side}\right)}\left(\tau ,\rho \right) & =\frac{\lambda^{2}\sigma^{0}}{16\pi^{3}c^{2}\tau^{3}}\frac{1}{\rho_{X}^{\prime }}\int_{-\infty }^{\infty }{\underline{G}}^{2}\left(\beta |\tau \right)\underline{b}\left(\rho -\beta |\tau \right)d\beta \\ & =\frac{\lambda^{2}\sigma^{0}\left(\tau \right)}{16\pi^{3}c^{2}\tau^{3}}\frac{1}{\rho_{X}^{\prime }\left(\tau \right)}\left[{\underline{G}}^{2}\left(\rho |\tau \right)\underset{\rho }{*}\underline{b}\left(\rho |\tau \right)\right]. \end{array}$$

If we note that the coefficients outside the brackets only depend on τ, we can simplify Equation (27):

(27) $$S_{h}^{\left(\mathrm{side}\right)}\left(\tau ,\rho \right)=f\left(\tau \right)\left[{\underline{G}}^{2}\left(\rho |\tau \right)\underset{\rho }{*}\underline{b}\left(\rho |\tau \right)\right].$$

The relationship in Equation (27) is a common model of the relationship between ICM spectrum, antenna pattern, and observed Doppler spectrum used in side-looking airborne radars (e.g., [54]); this derivation explicitly shows the conditions that are required for this model to be accurate.

We can also define a baseband analogue to the characteristic function of Equation (16) as:

(28) $$\underline{k}\left(\Delta t|\tau \right)=F_{\rho }^{-1}\left[\underline{b}\left(\rho |\tau \right)\right],$$

and note that for a side-looking radar:

(29) $$\begin{array}{cc} A_{h}^{\left(\mathrm{side}\right)}\left(\tau ,\Delta t\right) & =F_{\rho }^{-1}\left[S_{h}^{\left(\mathrm{side}\right)}\right]\\ & =f\left(\tau \right)F_{\rho }^{-1}\left[{\underline{G}}^{2}\left(\rho |\tau \right)\underset{\rho }{*}\underline{b}\left(\rho |\tau \right)\right]\\ & =f\left(\tau \right)F_{\rho }^{-1}\left[{\underline{G}}^{2}\left(\rho |\tau \right)\right]F_{\rho }^{-1}\left[\underline{b}\left(\rho |\tau \right)\right]\\ & =A_{h}^{\left(\mathrm{platform}\right)}\left(\tau ,\Delta t\right)\cdot \underline{k}\left(\Delta t|\tau \right), \end{array}$$

where $A_{h}^{\left(\mathrm{platform}\right)}$ is the autocorrelation due to platform motion only. Because $\underline{k}$ serves the function of windowing the correlation function (which in this case is also a covariance because the random process is zero mean) in Δt, it is referred to in the STAP literature as a covariance matrix taper (CMT) [27,28].

#### 2.5.3. Arbitrary Orientation

In general, for a narrow-azimuthal beamwidth antenna, the scattering function will be:

(30) $$S_{h}\left(\tau ,\rho \right)\approx S_{h}^{(\mathrm{no}}\;\mathrm{ICM})\left(\tau ,\rho \right)\;\underset{\rho }{*}\;\underline{b}\left(\rho |\tau \right),$$

where $S_{h}^{(\mathrm{no}}\;\mathrm{ICM})$ is the scattering function defined in Equation (21), which includes the Jakes spectrum peaks, and $\underline{b}$ is the ICM spectrum.

### 2.6. Output Time-Frequency Power Distribution

We will assume the matched-filter output is the correlation of *N* pulses against an infinite pulse train input, represented by the periodic ambiguity function (PAF) $|\chi_{NT}|$, given by [55]:

(31) $$|\chi_{NT}\left(\tau ,\rho \right)|=\left|\frac{1}{NT_{r}}\int_{0}^{NT_{r}}x\left(t\right)x^{*}\left(t+\tau \right)e^{j2\pi \rho t}dt\right|,$$

where x(t) is the normalized unit-energy input pulse train and $T_{r}$ is the pulse repetition interval (PRI). The pulse repetition frequency (PRF) $F_{r}=1/T_{r}$. The PAF has the following properties that are relevant to our application:

(32) $$|\chi_{NT}\left(\tau ,\rho \right)|=|\chi_{NT}\left(\tau +T_{r},\rho \right)|,$$

and:

(33) $$|\chi_{NT}\left(\tau ,\rho \right)|=|\chi_{1T}\left(\tau ,\rho \right)|\left|\frac{\sin N\pi \rho T_{r}}{N\sin\pi \rho T_{r}}\right|.$$

One important result from Green’s work [56] was that the time-frequency power distribution is proportional to the convolution of the ambiguity function with the WSSUS system scattering function:

(34) $$\begin{array}{cc} P(\tau ,\rho ) & =E_{x}S_{h}\left(\tau ,\rho \right)**{\left|\chi_{NT}\left(\tau ,\rho \right)\right|}^{2}\\ & =E_{x}\int \int S_{h}\left(\tau^{\prime },\rho^{\prime }\right){\left|\chi_{NT}\left(\tau -\tau^{\prime },\rho -\rho^{\prime }\right)\right|}^{2}d\tau^{\prime }d\rho^{\prime }, \end{array}$$

where $E_{x}$ is the energy of the pulse train. Note that since $|\chi_{NT}{|}^{2}$ is periodic along the τ axis, the result of the convolution integral of Equation (34) is periodic along this axis as well. If we assume the support of $S_{h}$ is limited to $\left[0,QT_{r}\right]$ for some positive integer *Q* along the τ axis, then the output power distribution can be written as a circular convolution:

(35) $$\begin{array}{cc} P(\tau ,\rho ) & =E_{x}\int_{0}^{QT_{r}}\int_{-\infty }^{\infty }S_{h}\left(\tau^{\prime },\rho^{\prime }\right){\left|\chi_{NT}\left(\tau -\tau^{\prime },\rho -\rho^{\prime }\right)\right|}^{2}d\rho^{\prime }d\tau^{\prime }\\ & =E_{x}S_{h}\left(\tau ,\rho \right)\underset{\tau }{⊛}\underset{\rho }{*}{\left|\chi_{NT}\left(\tau ,\rho \right)\right|}^{2}, \end{array}$$

where ⊛ represents circular convolution and * represents ordinary convolution. The problem was cast into this form so that discrete implementations can use the Fast Fourier Transform (FFT) to efficiently compute the periodic output power distribution along the τ axis without zero padding.

### 2.7. ICM Spectrum Characterization

#### 2.7.1. Maximum Entropy Prior

As it has been noted in Section 2.4, $\underline{b}\left(\rho |\tau \right)$ can be interpreted simultaneously as either (a) a PSD corresponding to a small patch of sea surface, or (b) a pdf of a random Doppler shift introduced by surface motion. Thus the spectrum characterization problem is reduced to a problem in prior probability density estimation.

Choosing a prior distribution is an important component of Bayesian estimation, and thus, there is a wide selection of literature available on the subject. If one has prior knowledge of the Doppler distribution from measurements or physical calculations, they can immediately compute the appropriate clutter taper using Equation (16). However, this is not usually the case, as the Doppler spectra for any given operating wavelength λ may depend on a multitude of factors, such as sea state, wind speed, temperature, salinity, etc.

In the absence of such detailed information, one tool for selecting an appropriate prior is the principle of maximum entropy (MaxEnt) [44]. It is based on the objectivist Bayesian philosophy that probabilities represent a state of knowledge rather than a degree of belief, and thus the selection of a prior distribution should be based on objective criteria, such as known expectations or known support of the random variable, in such a way that the amount of “assumed” information is minimized.

Formally, this is expressed as choosing some distribution function $\underline{b}\left(x\right)$ with support (a,b) of the random variable *X* that maximizes the differential entropy *H*:

(36) $$H\left(X\right)=-\int_{a}^{b}\underline{b}\left(x\right)\log\underline{b}\left(x\right)dx,$$

subject to the constraints imposed by the known “testable information” $F_{i}$:

(37) $$E\left[f_{i}\left(X\right)\right]=\int_{a}^{b}\underline{b}\left(x\right)f_{i}\left(x\right)dx=F_{i},\;i=1,\ldots ,n.$$

The solution to this optimization problem is found using the calculus of variations:

(38) $$\underline{b}\left(x\right)=c_{0}m\left(x\right)\exp\left(\sum_{i}\lambda_{i}f_{i}\left(x\right)\right),$$

where $c_{0}$ is a normalization constant, m(x) is a partition function that is constant in the support region x∈(a,b) and zero elsewhere, and $\lambda_{i}$ are the Lagrange multipliers. The shape of the resulting distribution depends on the amount of known “testable information”.

#### 2.7.2. Known Mean, Unknown Variance

We often know the wind speed $v_{w}$ and direction $\phi_{w}$ but no other information about the shape of the Doppler prior. If we start with the following assumptions:

- The mean wave speed (and hence mean Doppler shift) is proportional to the wind speed. For example, it is commonly assumed that the wave speed is 1/8th the wind speed [57], and
- The wind velocity vector has no *Z* component, and
- The waves move in the same direction as the wind,

then the following mathematical statements can then be constructed to express this testable information:

- $\mu_{\rho }\propto v_{w}\cos\theta \cos\left(\phi -\phi_{w}\right)$,
- $\rho \mathrm{sgn}\left(\mu_{\rho }\right)\in \left[0,\infty \right)$.

There are cases when these statements are not true, such as when there is a sudden change of wind direction, but for a fully developed wind-wave these are not particularly controversial statements [49]. The MaxEnt prior that satisfies these criteria can be shown to be:

(39) $$\underline{b}\left(\rho |\tau \right)=\frac{1}{|\mu_{\rho }\left(\tau \right)|}\exp\left(-\frac{\rho }{\mu_{\rho }\left(\tau \right)}\right)u\left(\rho \mathrm{sgn}\left(\mu_{\rho }\left(\tau \right)\right)\right),$$

where u(·) is the Heaviside step function. This is an exponential distribution with respect to the wind direction. The intuition behind this is that, while the mean Doppler shift will be proportional to the wind speed, there will be some wave components that move much faster, but none that move in the opposite direction of the wind.

#### 2.7.3. Known Mean and Variance

If the mean $E\left[\rho \right]=\mu_{\rho }$ and variance $E\left[{\left(\rho -E\left[\rho \right]\right)}^{2}\right]=\sigma_{\rho }^{2}$ of the Doppler frequency are known, then the MaxEnt prior is a truncated Gaussian:

(40) $$\underline{b}\left(\rho |\tau \right)=c_{0}m\left(\rho \right)\exp\left(-\frac{{\left(\rho -\mu_{\rho }^{\prime }\left(\tau \right)\right)}^{2}}{2\sigma_{\rho }^{\prime 2}\left(\tau \right)}\right).$$

In general $\mu_{\rho }\neq \mu_{\rho }^{\prime }$ and $\sigma_{\rho }^{2}\neq \sigma_{\rho }^{\prime 2}$ due to the truncation of the tails of the distribution by m(ρ). The Doppler spectra model given in §3.8.2 of [49] can be considered a version of this with a→−∞ and b→∞, in which case $\mu_{\rho }=\mu_{\rho }^{\prime }$ and $\sigma_{\rho }=\sigma_{\rho }^{\prime }$.

#### 2.7.4. Distribution Comparison

The claimed exponential shape of the MaxEnt Doppler spectrum may seem puzzling to experienced readers who have experience with Doppler spectra being commonly modeled as having a Gaussian shape. The reader is reminded that Equation (39) is not the spectrum the radar “sees”; the shape of the spectrum a (side-looking) radar will see is determined by the convolution (along ρ) of the Doppler spectra $\underline{b}\left(\rho |\tau \right)$ with the antenna pattern ${\underline{G}}^{2}\left(\rho |\tau \right)$, which is then convolved (along τ and ρ) with the waveform ambiguity squared $|\chi_{NT}{\left(\tau ,\rho \right)|}^{2}$. Each of these processing steps alters the resolution of the radar.

As an illustrative example, we will consider the Doppler spectra modeled in §3.8.2 of [49]. Since the radar is stationary, ${\underline{G}}^{2}\left(\rho |\tau \right)\propto \delta \left(\rho \right)$. The predicted spectrum in this scenario is described using the following parameters:

- $F_{r}=2$ kHz
- N=32 pulses
- Dolph-Chebyshev window with −55 dB sidelobes
- $\mu_{\rho }=62.5$ Hz
- $\sigma_{\rho }^{2}=20$ Hz^2^
- Overall clutter to noise ratio (CNR) = 20 dB

**Figure 5:**
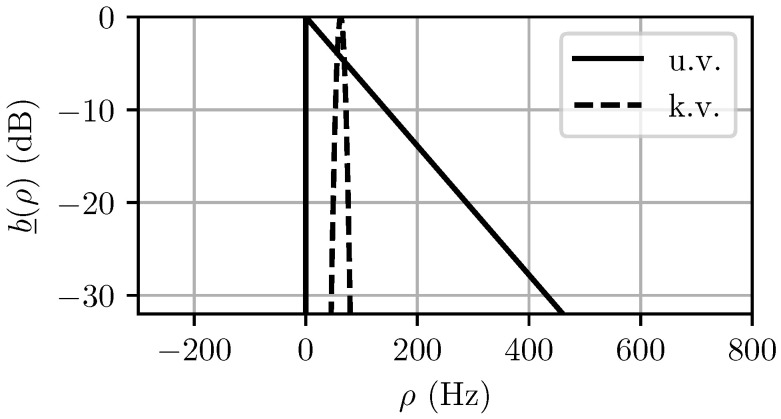
Maximum entropy (MaxEnt) Doppler priors for unknown variance (u.v.) and known variance (k.v.) cases. For the u.v. case, the MaxEnt prior is an exponential distribution, whereas for the k.v. case, the MaxEnt prior is a Gaussian. Note that additional knowledge of the variance significantly reduces the predicted spectrum width.

**Figure 6:**
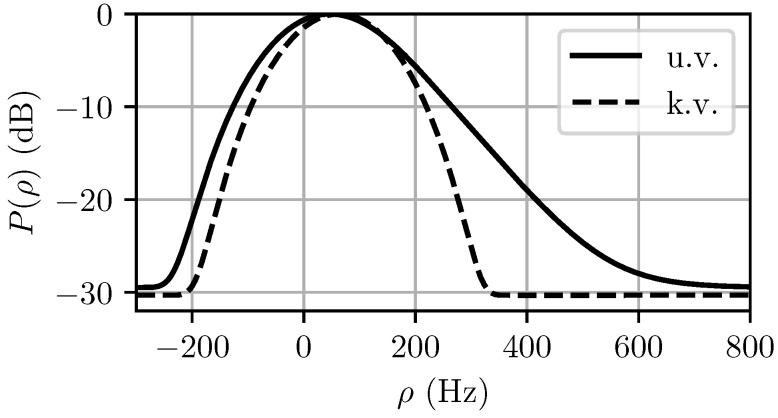
Output power distributions for unknown variance (u.v.) and known variance (k.v.) cases. Note that in this case the predicted spectrum when the variance is known becomes waveform-limited because $\sigma_{\rho }\ll F_{r}/N$.

The computed MaxEnt spectra for both the unknown and known variance cases are shown in Figure 5. The predicted power distribution $P\left(\rho \right)=|\chi_{NT}{\left(0,\rho \right)|}^{2}*\underline{b}\left(\rho \right)$ is shown in Figure 6. It can be seen in Figure 5 that the specification of the variance narrows the Doppler pdf $\underline{b}\left(\rho \right)$ significantly, but if too short of a CPI is used, as is the case in Figure 6, the observed spectrum after matched filtering will not change significantly.

### 2.8. Experimental Validation

#### 2.8.1. Doppler Spectrum Modeling

To validate our framework, we will compare the modeled clutter spectra to empirically measured spectra. For our comparison, we will use the Ingara dataset, which is a medium grazing angle dataset containing measured returns from an airborne radar flying in a circular path at a speed of approximately 200 knots, operating in spotlight mode (i.e., illuminating the patch of sea at the center of the circle.) Data were collected at grazing angles from 15 to 45 degrees. The radar operates at a center frequency of 10.1 GHz using a linear frequency modulated (LFM) waveform with a pulse width of 20 μs and a bandwidth 200 MHz, leading to a range resolution of about 0.75 m.

**Figure 7:**
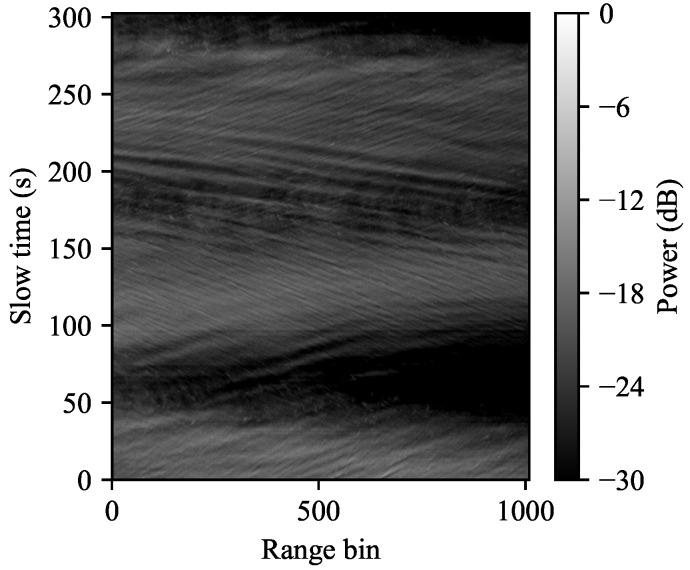
Ingara data from trial SCT04, flight F42, run 34,877. The Ingara radar contains 1024 range gates covering a range span of approximately 767 m, captured from an airborne radar operating in spotlight mode flying in a circular path at an altitude of 0.5 nmi and observing the ocean at a grazing angle of 15°. In this run, the plane completes approximately 1.4 revolutions of its circular flight path.

The data in the flight that we will use for comparison was collected from an altitude of 0.5 nmi with the plane flying in a circular path of radius 1.9 nmi, leading to a grazing angle of approximately 15 degrees. This run contains the measured radar IQ returns over a 305 second interval, or about 1.4 revolutions around the circular path. A plot of the downrange video at each slow time is shown in Figure 7. Close inspection of this picture shows that the individual wave crests and troughs are clearly visible.

**Figure 8:**
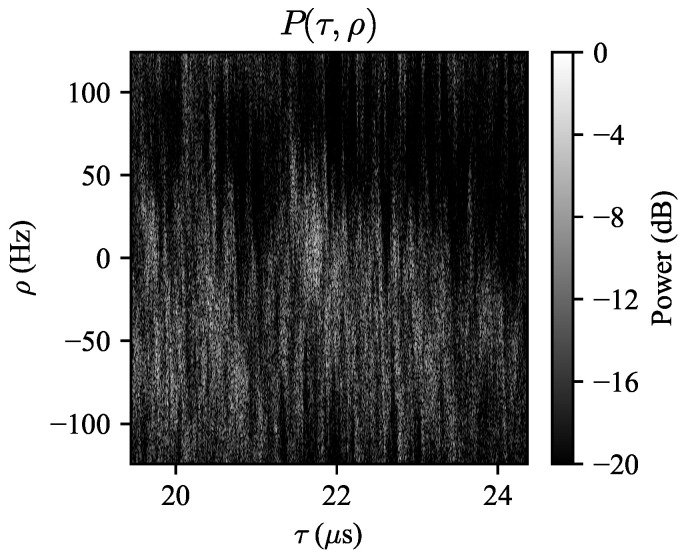
Range-Doppler map when the radar is looking in the downwind direction, obtained over a coherent processing interval (CPI) of 128 pulses (0.52 s). The wind speed was 8.5 m/s. Note that the Doppler shifts are biased towards negative frequencies. This bias could be due to wave motion away from the radar as well as antenna pointing errors.

**Figure 9:**
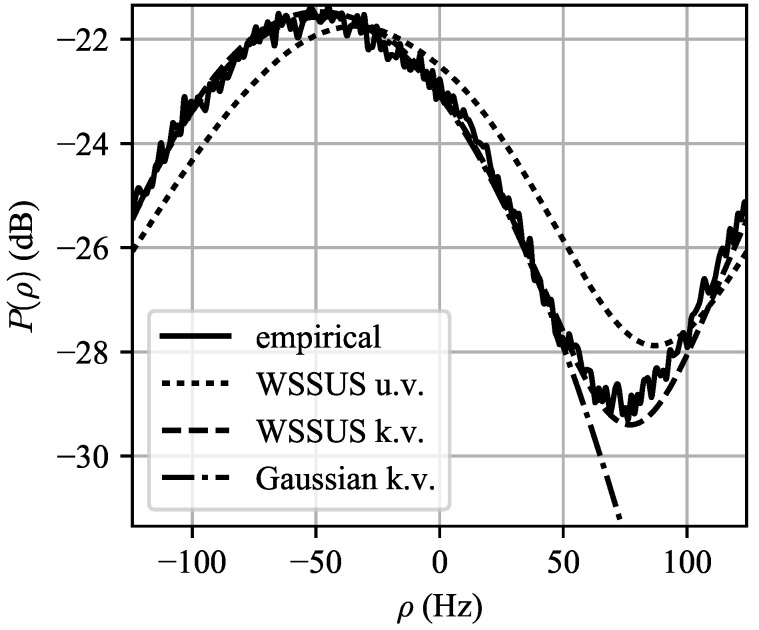
Empirical ensemble average spectrum plotted versus WSSUS predicted spectra for the unknown variance (u.v.) and known variance (k.v.) cases. As well, the “standard” Gaussian clutter spectrum when the variance is known is plotted for reference. It can be seen that the Gaussian spectrum model vastly underestimates the clutter floor caused by antenna and waveform sidelobes.

To test the ability of the WSSUS framework to predict Doppler spectra, we will compare the predicted spectra to the Doppler spectra observed while the radar is looking downwind. The observed range-Doppler map seen when the radar is looking downwind is shown in Figure 8. It can be seen that the spectrum is biased in the negative Doppler direction, as expected. Significant variation in the mean Doppler frequency in each range bin can be seen; this is likely due to the high range resolution of the radar. Because these minute fluctuations are essentially random and difficult to predict, we will take the ensemble average of the normalized spectra over τ. In this dataset, the elevation beampattern was removed as a preprocessing step, so it is expected that the Doppler spectra in each range bin will have similar statistics with the exception of path loss-induced amplitude decay. The ensemble average spectrum is shown in Figure 9, where it is labeled “empirical”.

Because the Ingara radar is side-looking and is flying at a constant altitude, we will use the simplifications of the WSSUS model in Section 2.5.2 to compute the scattering function $S_{h}$, which we will convolve with the ambiguity to obtain the delay-Doppler map P(τ,ρ). The azimuth antenna pattern and waveform parameterization were supplied with the dataset, so the only unknown in our prediction efforts is the ICM Doppler prior $\underline{b}\left(\rho |\tau \right)$.

We will use the MaxEnt procedure of Section 2.7.1 to estimate the ICM prior. For these models, it is necessary to predict mean and variance. Because the spectrum is periodic, we estimated the mean $\mu_{\rho }$ Doppler shift using the circular mean [45]:

(41) $$\mu_{\rho }\approx \frac{F_{r}}{2\pi }\arg\left(E_{P}\left[e^{j2\pi \rho /F_{r}}\right]\right),$$

where $E_{P}$ denotes expectation is taken with respect to the empirically observed spectrum P(ρ), normalized to unit area. From the data in Figure 9, $\mu_{\rho }$ = −47.9 Hz, which corresponds to a radial wave speed of −0.71 m/s. We will assume that $\mu_{\rho }$ accounts for both the mean ICM Doppler shift as well as antenna pointing errors.

For estimating the variance $\sigma_{\rho }^{2}$ of the prior, we need to account for the fact that, according to Equations (27) and (35), the spectral width is due to the convolution of the prior with the antenna with the waveform ambiguity, therefore each step “broadens” the spectrum. In the elementary case when all three functions are Gaussian, the observed spectral variance $\sigma_{\mathrm{obs}}^{2}$ is the sum of the component variances:

(42) $$\sigma_{\mathrm{obs}}^{2}=\sigma_{\rho }^{2}+\sigma_{a}^{2}+\sigma_{w}^{2},$$

where $\sigma_{a}$ and $\sigma_{w}$ are the RMS Doppler widths of the antenna pattern and waveform, respectively. This can be used to estimate the variance of the clutter prior:

(43) $$\sigma_{\rho }\approx \sqrt{\sigma_{\mathrm{obs}}^{2}-\sigma_{a}^{2}-\sigma_{w}^{2}}.$$

However, because the observed distribution P(ρ) and the waveform ambiguity cut $|\chi_{NT}{\left(0,\rho \right)|}^{2}$ are both periodic, the appropriate way to measure $\sigma_{\mathrm{obs}}$ and $\sigma_{w}$ are using the circular standard deviation [45]:

(44) $$\begin{array}{cc} \sigma_{\mathrm{obs}} & =\sqrt{-2\ln\left(\left|E_{P}\left[e^{j2\pi \rho /F_{r}}\right]\right|\right)}\\ \sigma_{w} & =\sqrt{-2\ln\left(\left|E_{{\left|\chi \right|}^{2}}\left[e^{j2\pi \rho /F_{r}}\right]\right|\right)}, \end{array}$$

where $E_{{\left|\chi \right|}^{2}}$ is expectation with respect to the normalized ambiguity squared.

The predicted spectrum using the WSSUS framework with the MaxEnt priors, labeled “WSSUS u.v.” for the unknown variance case, and “WSSUS k.v.” for the known variance case, as well a plot of the “standard” Gaussian Doppler spectrum model for P(ρ), are shown in Figure 9. It can be seen that both predicted spectra capture the shape of the empirical spectrum; the specification of the variance produces a spectrum that is within approximately 1 dB of the empirical spectrum at all frequencies. The Gaussian model for P(ρ) produces excelled agreement for in the main lobe but does not predict the presence of the sidelobe clutter floor of approximately –29 dB. This could lead to excessively optimistic predictions of radar performance in the sidelobe clutter region.

It is expected that for a side-looking radar in level flight the clutter Doppler spectrum will be centered at 0 Hz. The negative frequency bias of the observed Doppler spectrum in Figure 9 could be due to:

- Wave motion away from the radar, as described in Section 2.7.2, of approximately 0.71 m/s,
- Aircraft crabbing (i.e., translational motion in the *Y*-direction),
- Antenna pointing errors; using Equation (23) yields a pointing error of approximately 0.4 degrees,
- Some combination of the above effects.

#### 2.8.2. Validation of WSSUS Assumption

Another validation test that was performed using the Ingara dataset was to determine the validity of the WSSUS assumption. If the clutter impulse response h(τ,t) is non-WSS in *t*, then the autocorrelation $A_{h}\left(\tau ,\Delta t\right)$ would not just depend on the time-difference Δt, but also the absolute time *t*.

**Figure 10:**
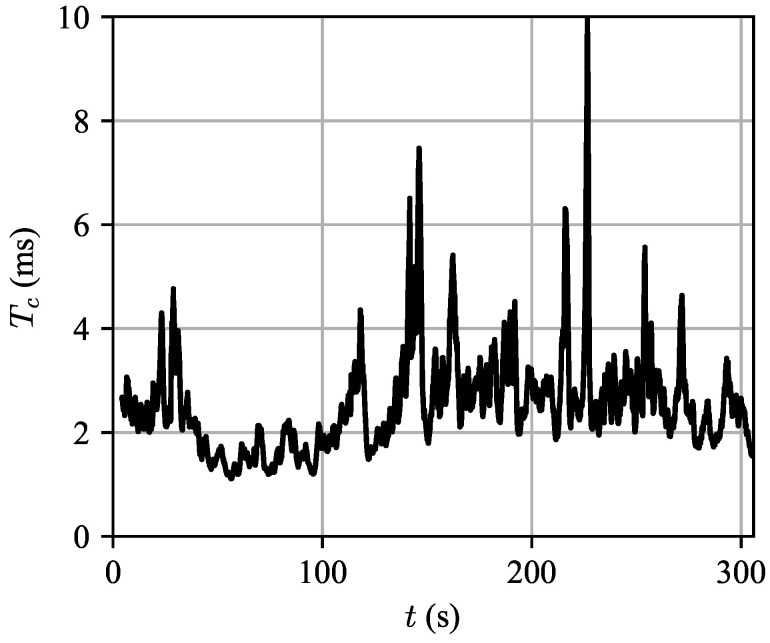
Clutter decorrelation times measured over time in range bin 1. Note that the decorrelation time remains roughly constant over several intervals much longer than a CPI. This provides support for the assumption that the clutter statistics are locally WSS for sufficiently long instants in time for the assumptions in Section 2.4 to apply.

To characterize the change in the autocorrelation over time, we computed the autocorrelation of the data over a sliding window of width 1 s over the entire record length of 305 s in range bin 1. The start time of each window was advanced by 10 ms, leading to a 99% overlap between windows. We will denote this function $A_{t}\left(\Delta t\right)$. To characterize the autocorrelation at each time instant *t*, we measure the decorrelation time $T_{c}$, defined implicitly by:

(45) $$\frac{A_{t}\left(T_{c}\right)}{A_{t}\left(0\right)}=e^{-1},$$

i.e., $T_{c}$ is the time it takes for the autocorrelation centered at time *t* to decay to 1/e from its peak value. The measured values of $T_{c}$ versus time are plotted in Figure 10.

We will use the stability of $T_{c}$ over time as a proxy to for the stationarity properties of the clutter. It is clear that the clutter is nonstationary over long periods of time, but for short instants of time it may be approximated as being locally WSS. Ironically, even researchers that study the nonstationary nature of clutter spectra must implicitly assume the clutter signal is locally WSS for them to be able to apply the Wiener-Khinchin theorem to estimate the clutter power spectrum in the first place! So the important question is not whether or not clutter is stationary, but how long does it remain locally stationary? Our modeling so far has assumed that the stationarity duration is larger than a CPI.

**Figure 11:**
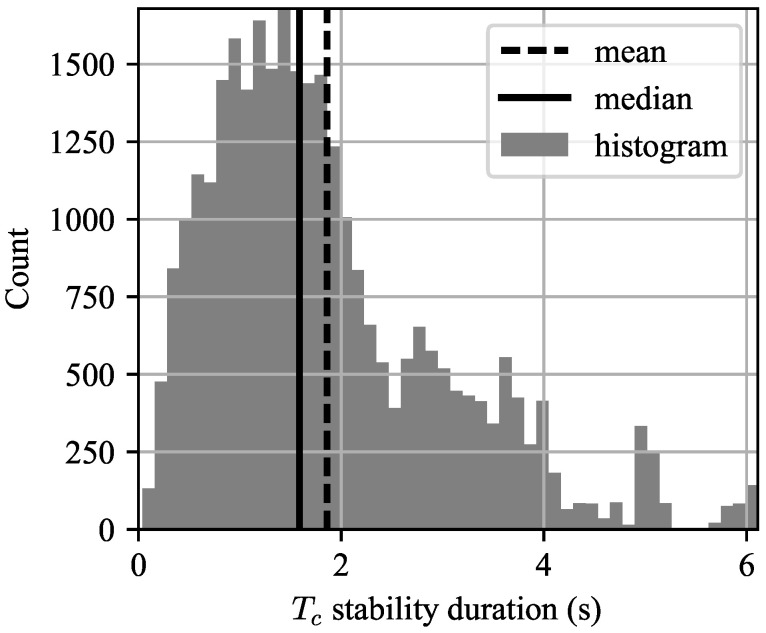
Histogram of stability durations. It can be seen that the decorrelation time $T_{c}$ is roughly constant over intervals much greater than one CPI for medium- and high-PRF waveforms.

To test this assumption, we created a statistic called the “stability duration” of $T_{c}$, which is defined as the region of time over which $T_{c}$ changes less than X%. (We used 10 percent, but this is admittedly arbitrary.) Because $T_{c}=T_{c}\left(t\right)$ is itself a function of time, the stability duration was computed relative to the value of $T_{c}\left(t\right)$ at each timestep. A histogram of the stability times over the entire record length is shown in Figure 11. It can be seen that the median stability time is about 1.5 s, with the mean being even higher. Thus for any waveform with a PRI on the order of one millisecond or less (i.e., PRF > 1 kHz, which is very common) and integrating less than 1000 pulses, this is a perfectly reasonable assumption. This assumption may be violated for low-PRF waveforms with long integration times, such as those used in surveillance radars.

## 3. Discussion and Conclusions

Effective suppression of unwanted sea clutter returns requires an accurate characterization of their spatio-temporal statistics. We have created a WSSUS model for sea clutter that allows for the prediction of the clutter range-Doppler spectrum and provided a mechanism by which internal clutter motion may be accounted for and quickly estimated. Validation against the Ingara medium grazing angle dataset shows agreement between the WSSUS model and the ensemble average spectrum to within 1 dB at all frequencies.

Future work could apply this model to developing signal processing techniques such as adaptive transmit waveform design and improved MTI filtering to optimize signal detection in the presence of heterogeneous clutter.

## 4. Materials and Methods

The Ingara dataset, which was used for all experiments in this paper, is not publicly available; access is controlled by the Australian Defence Science and Technology (DST) Group [46]. Data analysis methods are described fully in the paper.

References
